# The need for methodological pluralism in epidemiological modelling

**DOI:** 10.1016/j.gloepi.2024.100177

**Published:** 2024-12-10

**Authors:** Pieter Streicher, Alex Broadbent, Joel Hellewell

**Affiliations:** a Centre for Philosophy of Epidemiology, Medicine, and Public Health, University of Johannesburg, South Africa; b Centre for Philosophy of Epidemiology, Medicine, and Public Health, Durham University, United Kingdom; c EMBL European Bioinformatics Institute, United Kingdom

## Abstract

During the Covid-19 pandemic, the best-performing modelling groups were not always the best-resourced. This paper seeks to understand and learn from notable predictions in two reports by the UK's Scientific Advisory Group for Emergencies (SAGE). In July 2021, SAGE reported that, after the upcoming lifting of restrictions (“Freedom Day”) cases would “almost certainly remain extremely high for the rest of the summer” and that hospitalisations per day would peak between 100 and 10,000. Cases were not “extremely high” and began to decline, while hospitalisations initially lay outside (above) SAGE's confidence bounds, and only came within the expected range when the upper and lower bound moved so far apart as no longer to be useful for policy or planning purposes. The second episode occurred in December 2021, when SAGE projected 600–6000 deaths per day at peak in the scenario where restrictions remained as they were (referred to as “Plan B"). In the event, restrictions did not change, and deaths peaked at 202, well below the lower bound, even though this spanned one order of magnitude. We argue that the fundamental problem was over-reliance on mechanistic approaches to disease modelling, and that a methodologically pluralist approach would have helped. We consider various ways this could have been done, including evaluating past performance and considering data from elsewhere. We show how the South African Covid-19 Modelling Consortium performed better by learning from experience and using multiple methods. We conclude in favour of methodological pluralism in infectious disease modelling, echoing calls for methodological pluralism in recent literature on causal inference.

In this article, we seek the lessons of two high-profile episodes of poor predictive performance by the UK's Scientific Advisory Group for Emergencies (SAGE). These episodes occurred in July and December 2021, concerning Delta and Omicron variants respectively. SAGE published projections about scenarios which were then realised by policy decisions, effectively rendering these scenario-projections into forecasts or predictions about the actual future course of events. In both cases, these predictions proved to be not fit for purpose. In both cases, the range of possible outcomes predicted was too wide to be useful for planning or decision purposes; and in both cases, there were (nevertheless) significant inaccuracies, i.e. discrepancies between what was predicted and what happened. It is important to understand how this happened, not just for the sake of British policy, but also because SAGE included strong representation from the World Health Organisation (WHO)’s sole collaborating centre for infectious disease modelling, hosted at Imperial College London. The UK's SAGE responded to scientific advice that was also globally influential, and represents a well-documented context in which to review that advice for learnings.

The authors would like to emphasise that our criticism is aimed at a specific modelling approach used with the shared goal of improving our ability to use models and data to inform decision making during a major crisis. We accept that those involved in SAGE were sincere, committing huge effort with little reward during a very challenging and extended period. There is an enormous chasm between using modelling in a long-term research context and using modelling during crisis management. Nonetheless, it is important to identify challenges and, where appropriate, errors, so as to learn from them.

In this piece, we argue that the fundamental problem behind SAGE's performance in these two episodes was relying heavily on one approach to predictive modelling while neglecting others. We will further argue that this strong preference for a certain methodological approach resulted in a lack of evaluation of model performance and of data and approaches used by other countries. We compare the South African Covid-19 Modelling Consortium (SACMC) which recognised problems and responded with not only new methods but a pluralistic approach to deploying them, and which performed much better than SAGE (despite being much less well resourced) in the Omicron wave.

SAGE focused on *mechanistic* models, which seek to simulate dynamics of transmission in order to predict the course of the epidemic. These have become much more powerful in recent years due to technological and mathematical developments, enabling sophisticated microsimulations with extraordinary power to model alternative scenarios. However, this power requires extraordinary data, and sufficient data which not always available during the Covid-19 pandemic. In the absence of sufficient, reliable data, assumptions or data errors, even if small, can produce significant errors in the context of an exponentially growing pandemic even if the models themselves are sound.

One way to mitigate this risk of error is to use simpler *descriptive* or statistical approaches to modelling, *alongside* (not necessarily instead of) mechanistic approaches. Descriptive approaches seek an accurate representation of current trends and develop forecasts on that basis. SAGE did not refer to this kind modelling in its published reports, and on occasion, some modellers criticised teams who used descriptive approaches, and participated in attacks in the media [[1], [2], [3]].

The use of two or more approaches to a problem is known as *methodological pluralism* and has also been advocated in the context of causal inference [4,5]. If different methods yield different conclusions, at least one of them must be wrong, prompting further checking. However, if a conclusion (in this case, a prediction) is arrived at using two (or more) different methods, there is more reason for confidence than can be derived from just one method. This is also sometimes known as “triangulation” and has been likened to a crossword puzzle [6].

Another reason to use multiple methods is that different methods are better for different purposes. Descriptive models are responsive to current data, making them particularly useful for short-term forecasts. They are thus used for planning purposes and resource allocation. They are, on the other hand, usually less useful for predicting the effect of interventions or other changes, where mechanistic models are more useful. This means the latter are commonly used for informing decision-making. The two kinds of models are both useful and may be used together to supplement and cross-validate each other. However, in July and December 2021, SAGE reported on only a handful of models (four and two respectively), all of the mechanistic kind, and based its recommendations solely on these. The result on both occasions was error. We will show that the errors could have been avoided by the adoption of a methodologically pluralistic approach.

## Two examples of poor predictive performance

The first of the two episodes we focus on occurred in July 2021, when SAGE produced a report ahead of planned easing of restrictions later that month (known as “Freedom Day”) [7]. The report predicted prevalence would “almost certainly remain extremely high for at least the rest of the summer”. In reality, cases did not “remain extremely high”, as SAGE was “almost certain” they would. They had just peaked at 44,876 per day (7-day average peak) on 16 July 2021, declining to a level of around 30,000 per day from August to November 2021. While “extremely high” is vague, a contemporaneous reader would not expect these numbers, nor the declining trajectory, especially given the modifiers “extremely” and “almost certainly”.

A contemporaneous reader might also have been influenced by a SAGE SPI-M member who said in a television interview that it was “almost inevitable” cases would rise to 100,000 a day, the uncertainty being whether they would rise to 200,000 or higher [8]. While this was not an official SAGE statement, it was not contradicted by SAGE, and the interview was obviously prompted by SAGE's report, and naturally understood as a commentary upon it by a prominent member and influential public figure. If SAGE significantly disagreed with a public statement by one of its members, it could, should, and probably would have issued a statement to this effect.

The second episode occurred in December 2021, when SAGE issued a consensus statement projecting between 600 and 6000 deaths daily in England in a scenario in which restrictions were not significantly tightened [9]. The political leadership did not alter restrictions, and thus the scenario was actualised, and the counterfactual projection became an actual prediction. Daily deaths peaked at 202 (7-day average peak), three times lower than the lower bound of the Plan B scenario and thirty times lower than the upper bound of the Plan B scenario.

The term “prediction” is sometimes analysed further by distinguishing projections and forecasts. A projection concerns what would happen in a certain scenario, which may be counterfactual, while a forecast concerns what will actually happen. However, this distinction becomes redundant where the scenario of a projection is actualised, when that projection becomes a forecast. That is what happened in these instances, enabling the evaluation of model performance.

In addition to being insufficiently accurate, both these reports were excessively vague*.* Modelling is always done with a purpose, and the confidence intervals of these predictions were excessively wide for either decision-making or planning purposes. That is to say, even where the predicted range of possible outcomes included actual outcomes, the range of possible outcomes was too wide to guide either a decision or preparation. In July 2021, SAGE predicted daily hospitalisations spanning 100–10,000: two orders of magnitude within one month after the lifting of restrictions. This included the actual outcome, a peak around 1000 per day. However, neither policy decision nor planning is usefully informed by such a large range, and could have gone significantly awry even with the benefit of these projections. (Imagine planning for a party with between 100 and 10,000 guests.) Acknowledging this uncertainty, SAGE advised preparing for the upper limit, which would have meant over-preparing by 9000 beds. Had this advice been followed, a serious misallocation of resources would have resulted, and policy turns to science precisely to minimise the misallocation of finite resources.

**Fig. 1:**
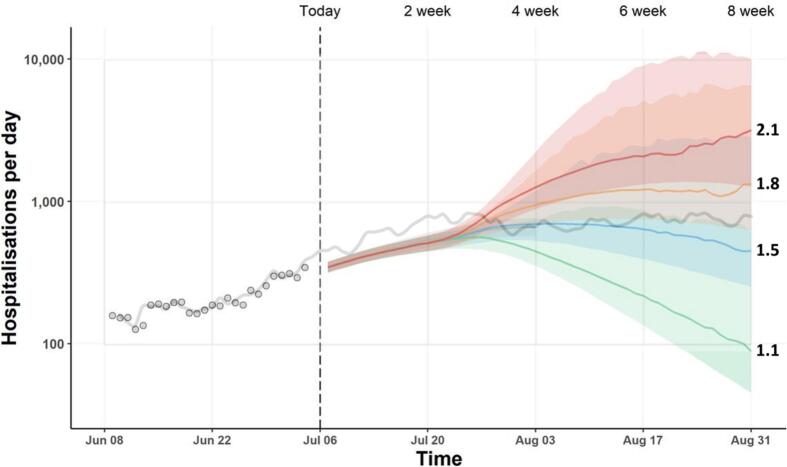
SAGE Report from July 2021, Fig. 1, with actual hospitalisations superimposed (grey line).

It is interesting that SAGE's confidence bounds were contrastingly too narrow in the period between “Freedom Day” and the reduction of restrictions having an effect. Data for much of this period (i.e. the weeks before Freedom Day) was available, so predictions of hospitalisations were forecasts based on available data, not projections about possible scenarios. Fig. 1 shows actual hospitalisations superimposed upon SAGE's projections. Actual hospitalisations lie above SAGE's upper bound until around the end of July, at which point they move within the upper and lower bounds. (It is important to note the logarithmic scale of this figure: although the actual curve is around the middle of the image, this implies actuals remained more than ten times below the upper bound at peak.) While the actual numbers fell within what was predicted, this was only the case after the upper and lower bounds diverged significantly. For the period of time before, i.e. the period for which actual data was available, actual hospitalisations were significantly above SAGE's forecasts based on that data. SAGE was actually too confident about what would happen prior to the point at which Freedom Day had an effect, as well as too vague about what would happen afterwards.

In December of that year, when predicting the effect of Omicron under light restrictions, SAGE again produced a wide confidence interval, one order of magnitude. Again, for the purposes of informing either policy-making or planning, this is a very large difference at the scales involved: 6000 deaths a day is much more than 600 in absolute terms. Of course, as we have already pointed out, the prediction was also inaccurate despite its wide confidence intervals, so even preparing for the lower bound would have been preparing for something three times worse than what actually happened. (Thus while the confidence interval was too wide to be useful for policy, it was not wide enough in a statistical sense, since it did not include the truth.)

A common explanation of both inaccuracy and vagueness in modelling outputs was that for each scenario, a worst-case outcome was presented rather than a likely outcome. However, this is not a strong explanation. The lack of guidance concerning confidence within the upper and lower bounds is problematic in itself. If the upper bounds of both intervals represented reasonable worst-case scenarios, it would have been reasonable to expect the likely outcome somewhere in between these wide confidence intervals, and if that was not reasonable, then this fact should certainly have been indicated at the time. Moreover, given what actually came to pass, it is prima facie implausible that the upper bounds represented reasonable worst case scenarios in either case. The implausibility is compounded when we consider what descriptive models would have predicted, in the next section.

The second of these episodes produced public furore [[10], [11], [12], [13]]. But public furore is not usually a good means of learning lessons, and rather inhibits open evaluation by modellers. Accusations of a political or personal kind tend to obscure rather than illuminate the fundamentally important question: How could what is arguably the most powerful modelling community in the world (which included the WHO's collaborating centre on infectious disease modelling) get it so wrong? This is not a rhetorical question, but a real and important one. Unless it is answered, this kind of error will probably happen again.

## Complementary forecasts from descriptive models

**Fig. 2:**
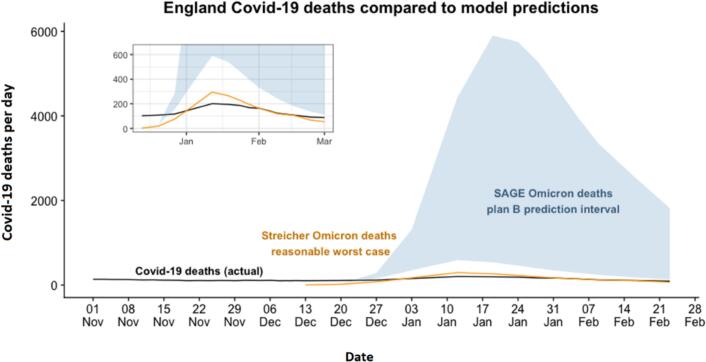
Projected deaths from SAGE (blue band), the authors' contemporaneous model (brown line) of Omicron under a continuation of then-current light restrictions, and actual deaths (black line). (For interpretation of the references to colour in this figure legend, the reader is referred to the web version of this article.)

The most obvious way descriptive models can usefully accompany mechanistic models is by producing complementary contemporaneous forecasts. In December 2021 a new variant, Omicron, had arrived and was spreading in the UK. According to data from South Africa, it had already peaked there, and was considerably milder than previous variants. It was therefore reasonably straightforward to fit a descriptive model to the available UK data, making adjustments for factors such as population age and prior immunity levels. At the time, our own contemporaneous model taking data from Gauteng (SA's most densely populated province) told us to expect a worst-case scenario of 350 deaths per day for the UK, derived from peak confirmed C-19 deaths in Gauteng, increased by a factor of 2.4× (to account for 50 % under-ascertainment of deaths in Gauteng, where deaths outside hospital were not reliably recorded) [14]. This amounts to 295 per day for England on a simple proportion basis. Actual deaths in England peaked at 202 per day (Fig. 2). A formalised approach has since been developed through retrospective analysis by other authors [15].

SAGE had this to say about South Africa: “There are early indications for shorter lengths of [hospital] stay in South Africa [implying lower severity], however, it is unknown whether such observations would be seen in the UK, given differences in population demographics, COVID-19 epidemic timing and variant composition to date, vaccination types and programmes, health care systems, and so on.” Instead, for its December 2021 forecast, SAGE based itself on two models from UK universities, both using similar mechanistic approaches.

SAGE is right to point out differences between South Africa and the UK. However, this is not sufficient reason to ignore data from another country, especially if it is the only data available at the time. In the absence of anything better, it is more appropriate to intelligently evaluate the differences between the two countries, and to consider their likely relevance. In this case, there was information available about hospital fatality rates in the two countries in previous waves. Moreover, a breakdown between public and private hospitals in South Africa (with different levels of care) was available, and in different provinces. SAGE's predicted upper bound HFR of 60 % (6000 deaths per day for 10,000 hospitalisations) would have represented a very wide deviation from that observed in private hospitals in Gauteng, South Africa's most populous province (and in others, but we are here illustrating an approach that might have been taken, not undertaking a full-blown analysis). A national HFR of 60 % would also have been much higher than South Africa's overall HFR of 9 %, which included less well-resourced public hospitals. In the event, England's HFR was 10 %.

Even setting aside the South African data, a review of HFR in previous England waves would have shown the upper bound of 60 % to be extremely divergent from previous waves. HFRs in England declined consistently from the first wave (43 %), to the Alpha wave (32 %) and to the Delta wave (16 %). Given widespread vaccination and previous exposure, a jump to 60 % would have represented a much more dangerous variant than anything previously seen.

Note that we are not recommending that SAGE should have based itself on such an analysis *instead* of the models it actually used, but that it should have done *both*, accompanied by the kind of qualitative reasoning from multiple perspectives that we have just sketched*.* If SAGE had used multiple methods, especially where they applied to multiple data sources, and published the results alongside the mechanistic modelling output, and *then* tried to explain why the South African experience was irrelevant, it would almost certainly have devoted more than one sentence to the matter. It would also have needed to explain fully why Omicron would be so much more dangerous in the UK than previous variants. More likely, it would have sought further information, and probably modified its results in the direction of greater accuracy.

## Prospective and retrospective evaluation

When Omicron emerged, the example of South Africa made it possible to develop a descriptive model about the consequences of persisting with light restrictions in the UK. In July 2021, there was nothing similar that SAGE might have used to develop a descriptive model of the consequences of Freedom Day. However, SAGE had had the opportunity to assess the performance of the approaches it was using. Forecasts from SAGE's models, constructed retrospectively, could have tested whether these models made good forecasts of disease outcomes when real changes in exogenous factors (such as population movement) were used as inputs. (SAGE could also have considered the performance of its Freedom Day predictions in the two weeks immediately following, when hospitalisations were outside (above) upper confidence bounds of forecasts based on the recent past.)

Descriptive models could have been used to perform this exercise for occasions where policy changes had been implemented in the past, by developing counterfactual predictions about what would have happened, if a policy change had not been implemented when it was – i.e. if things had gone on as they were. To justify their continued use, mechanistic models might be expected to at least match, if not outperform, simple descriptive models applied to past scenarios. However, this kind of exercise was never performed by SAGE. As one of the authors of this paper, and also a member of the modelling team at the London School of Hygiene and Tropical Medicine, put it later (just before the Omicron episode):

It's worth thinking about how this process must have looked to an enthusiastic outsider: modellers have continued to deploy their essentially unverified models again and again at each new wave and reopening stage, seemingly oblivious to the accuracy of previous predictions. [16]

Trying to determine the predictive performance of a model using retrospective forecasts is important, since the accuracy of counterfactual projections also determines the accuracy of the estimated effect of interventions. The team that produced the influential report published 16 March 2020 by Imperial College London [17], also conducted the most prominent evaluation of the effectiveness of the first UK lockdown [18]. They used similar methods (mechanistic modelling), and did not apply a descriptive model to evaluate their previous predictions and assumptions about a no-lockdown scenario.

This was also not done a year later when the same team produced a fresh assessment, again using similar mechanistic modelling approaches [19]. This assessment produced a much reduced estimate of lockdown effect, attributed to anticipatory behaviour change. The finding and explanation might have excited more scrutiny had they appeared a year before. SAGE was certainly aware of work in May 2020 (although not published until 2021, after a lengthy peer review journey) suggesting that infections peaked before lockdown, and using different methods to do so [20].

In any case, there is a difference between assessing the effectiveness of lockdown and assessing the predictive performance of a model. It is as if the assessment of lockdown effectiveness stood in as a proxy for evaluating the modelling that informed the decision, in place of direct assessment of the performance of the underlying modelling.

**Fig. 3:**
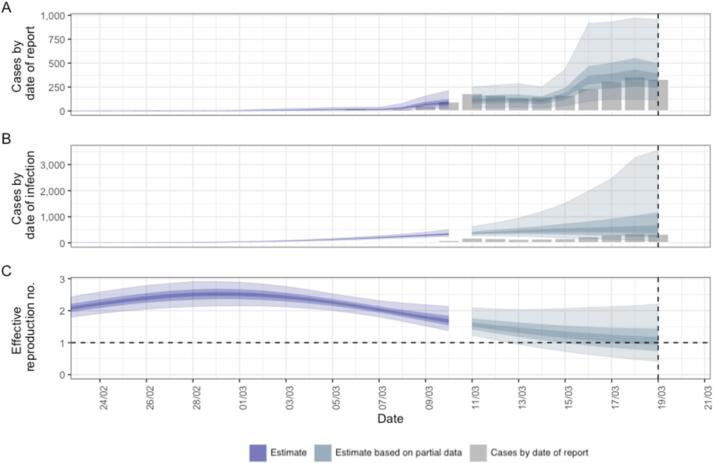
Results of fitting a current model for estimating R_t_ to reported case data for London up to 20th March 2020, just prior to the first UK lockdown. The model is parameterised using a generation time distribution [23] and incubation period distribution [24] that were not available in March 2020. Data: https://epiforecasts.io/EpiNow2/

To our knowledge, the exercise of retrospectively fitting a descriptive model to data available in early March 2020 has still not been done since. We therefore retrospectively applied a current descriptive model for estimating *R*_*t*_ (EpiNow2), representing our best *current* knowledge, to the situation as it was shortly before the first lockdown in London. This tells us what, according to our best current knowledge, was likely to have happened if things had gone on as they were before lockdown on 23 March 2020. (Whether or not this is a plausible scenario does not matter, because we are evaluating another model's claims about the same scenario; and it was these claims that informed policy decisions at the time.) The best current model suggests that *R*_*t*_ was below 1 in London before the lockdown of 23 March 2020 was introduced (Fig. 3). Post-hoc analysis based on deaths, hospital data and testing data done by statisticians [20,21], and later also by ICL [22], confirms this. If the epidemic had already begun to decline when lockdown regulations came into force, then that event could not have caused the peak. Anticipatory behaviour change might explain this, but another possibility is that the predictions in the report published on 16 March 2020 were wrong, and the methods that produced them inaccurate.

Our conclusion concerns model performance and not lockdown effectiveness or policy correctness. This is an exercise to see whether alternative methods supply prima facie corroboration for the modelling approach that SAGE relied on throughout the pandemic. That this corroboration exercise was never deemed worthwhile suggests that there was a high degree of confidence in the methods being used. A more pluralistic methodological approach would probably have tempered this confidence.

## Looking into the future

It is easy to blame poor predictive performance on the intrinsic difficulty of prediction, or to see the limitations of a method as insurmountable epistemic limitations. This is harder if others made better predictions in similar circumstances [25,26]. The SACMC initially produced short-term forecasts that were inaccurate [27]. During the second wave, the SACMC “made the difficult decision not to produce model-based projections”, instead developing a “set of metrics that could detect and monitor the second wave” [28]. By the third wave, the SACMC was more confident, due to experience gained from monitoring the second wave, and produced both longer term forecasts based on mechanistic models alongside short-term forecasts based on descriptive approaches. The SACMC's cross-referencing of different approaches contrasts favourably with SAGE's confidence in a single approach.

The SACMC's approach can be described as methodologically pluralistic. The methodology it began with performed poorly, and therefore methods were refined and alternatives were developed. The SACMC adopted the practice of publishing results of forecasts alongside its projections of the results of interventions. We attribute its success in Omicron to these practices. The policy decision to maintain existing restrictions was a high-stakes decision, since an unnecessary tightening would have caused significant hardship for many very poor people, while an erroneous decision not to tighten could have led to significant Covid-19 mortality. SAGE appeared not only to set aside South African data, but also to neglect the achievement of the South African modellers who made the correct call in a high-stakes context. Had SAGE considered how the SACMC arrived at its conclusion, it might have seen the potential value of using a plurality of methods.

Every method has its drawbacks. Methodological pluralism has been advocated in the contexts of causal inference [5] and health complexity [29]. It can be promoted by including people with different expertise in advisory committees (for example, by including statisticians with forecasting expertise on SAGE-SPI-M). Reliance on a single method, no matter how powerful, leads to neglect of important evidence and to error. The advent of a powerful new method, be it randomized controlled trials, potential outcomes frameworks for causal inference, or simulation modelling on powerful computers, can create an excess of enthusiasm. A person with a hammer sees a screw as a nail. In South Africa, modelling accuracy improved substantially as the importance of descriptive models was recognised. However, where there was a strong commitment to a single methodological approach, avoidable errors persisted. As infectious disease modelling matures, it must become more methodologically pluralistic if it is to become more predictively accurate.

## CRediT authorship contribution statement

**Pieter Streicher:** Writing – review & editing, Investigation, Formal analysis, Conceptualization. **Alex Broadbent:** Writing – original draft, Investigation, Conceptualization. **Joel Hellewell:** Writing – review & editing, Methodology, Formal analysis, Conceptualization.

## Declaration of competing interest

The authors declare that they have no known competing financial interests or personal relationships that could have appeared to influence the work reported in this paper.
